# Vitrectomy with or without internal limiting membrane peeling for idiopathic epiretinal membrane: A meta-analysis

**DOI:** 10.1371/journal.pone.0179105

**Published:** 2017-06-16

**Authors:** Wei-Cheng Chang, Chin Lin, Cho-Hao Lee, Tzu-Ling Sung, Tao-Hsin Tung, Jorn-Hon Liu

**Affiliations:** 1 Department of Ophthalmology, Cheng Hsin General Hospital, Taipei, Taiwan; 2 School of Public Health, National Defense Medical Center, Taipei, Taiwan, Republic of China; 3 Graduate Institute of Life Sciences, National Defense Medical Center, Taipei, Taiwan, Republic of China; 4 Department of InternalMedicine, Tri-Service General Hospital, National Defense Medical Center, Taipei, Taiwan, Republic of China; 5 Department of Medical Research and Education, Cheng Hsin General Hospital, Taipei, Taiwan; 6 Faculty of Public Health, School of Medicine, Fu-Jen Catholic University, Taipei, Taiwan; International University of Health and Welfare, JAPAN

## Abstract

**Background:**

Studies on vitrectomy with and without internal limiting membrane (ILM) peeling for idiopathic epiretinal membrane (ERM) have yielded uncertain results regarding clinical outcomes and recurrence rates.

**Objective:**

To compare the clinical outcomes of vitrectomy with and without ILM peeling for idiopathic ERM.

**Methods:**

Databases, including PubMed, Embase, Cochrane, Web of Science, Google Scholar, CNKI databases, FDA.gov, and ClinicalTrials.gov, published until July 2016, were searched to identify studies comparing the clinical outcomes following vitrectomy with ERM and ILM peeling and with only ERM peeling, for treating idiopathic ERM. Studies with sufficient data were selected. Pooled results were expressed as mean differences (MDs) and risk ratios (RRs) with corresponding 95% confidence intervals (CI) for vitrectomy with and without ILM peeling with regard to postoperative best corrected visual acuity (BCVA), central retinal thickness (CRT), and ERM recurrence rate.

**Results:**

Eleven retrospective studies and one randomized controlled trial involving 756 eyes were identified. This demonstrated that the postoperative BCVA within 12 months was significantly better in the non-ILM peeling group (MD = 0.04, 95% CI: 0.00 to 0.08; *P* = 0.0460), but that the patients in the ILM peeling group had significantly better postoperative BCVA after 18 months (MD = −0.13, 95% CI: −0.23 to −0.04; *P* = 0.0049) than did those in the non-ILM peeling group. The non-ILM peeling group exhibited a higher reduction in postoperative CRT (MD = 51.55, 95% CI:−84.23 to −18.88; *P* = 0.0020) and a higher recurrence rate of ERM (RR = 0.34, 95% CI:0.16 to 0.72; *P* = 0.0048) than did the ILM peeling group. However, the improvement rates of BCVA (RR = 1.03, 95% CI:0.72 to 1.47; *P* = 0.8802) and postoperative CRTs (MD = 18.15, 95% CI:−2.29 to 38.60; *P* = 0.0818) were similar between the two groups.

**Conclusions:**

Vitrectomy with ILM peeling results in better visual improvement in long-term follow-ups and lower ERM recurrence rates, and vitrectomy with only ERM peeling is more efficacious in reduction of CRT than is vitrectomy with ILM peeling.

## 1. Introduction

An epiretinal membrane (ERM), also known as a macular pucker, is a condition affecting the avascular fibrocellular membrane over the central macular area between the vitreous and internal limiting membrane (ILM). Its pathogenic mechanism has an unknown etiology and can be idiopathic or secondary to other ocular diseases, trauma, or previous intraocular operation. The incidence of idiopathic ERM reportedly ranges from 2% in patients younger than 60 years to 12%–20% in those older than 70 years [1]. It may reduce visual acuity (VA) and cause micropsia, macropsia, monocular diplopia, metamorphopsia, or even progressive vision loss [2]. Some hypotheses of the pathogenesis of ERM have involved postulating the proliferation of fibroblasts, glial cells, and astrocytes after ILM disruption, following posterior vitreous detachment [3, 4]. Sebag et al. involve speculating that a residual posterior vitreous cortex (vitreouschisis), attached to the macula during the liquefying process of the vitreous body, may play a role in ERM development [5]. Kishi and Shimizu reported that premacular vitreous cortex, which forms the posterior wall of the premacular liquefied pocket, plays a key role in the development of idiopathic preretinal macular fibrosis in eyes with or without posterior vitreous detachment [6].

Pars plana vitrectomy with membrane peeling has been effectively used for the surgical treatment of ERM since 1978 [7]. A high visual improvement rate, up to 90%, and a recurrence rate of 1%–16% have been reported after successful surgery [3, 8–11]. Currently, the surgical methods for membrane peeling have evolved because of the use of dyes. Triamcinolone stained the cortical vitreous and ERM although not the ILM [12], whereas indocyanine green (ICG), trypan blue, and brilliant blue G (BBG) were used to stain the ILM [13]. Some ILM peeling reports have revealed results such as improved VA outcomes, lower recurrence rates, and reduced retinal striae [14, 15]. The ILM peeling procedure is increasingly being used by retinal surgeons from 25% in 2008 to 44% in 2010 [16]. Although an increasing number of vitrectomy with ILM peeling has been reported, ILM peeling is believed to cause functional and mechanical damage to the Muller cells because the ILM is the basal lamina connected to the end feet of the Muller cells [17–19]. Whether to consider peeling of the ILM a surgical method for treating idiopathic ERM disease continues to be debated among vitreoretinal surgeons.

Because of the uncertainty of the effectiveness of ILM peeling in idiopathic ERM surgery, many studies have investigated the single peeling (only ERM) and double peeling (ERM and ILM peeling) surgical techniques for idiopathic ERM in recent 20 years since 1993 [15]. Most studies were retrospective comparative studies; however, only one new randomized control trial with a sufficiently large sample size and appropriate design was published in 2016 [20]. Until now, only one meta-analysis has enrolled eight retrospective studies to compare the effectiveness of pars plana vitrectomy with and without ILM peeling for idiopathic ERM removal [21]. The results reported by Liu et al. revealed uncertainty in postoperative VA outcomes and nonsignificant differences in ERM recurrence rates in the meta-analysis because of the limited sample size. In our study, we attempted to include retrospective studies published in recent years and recruited the results of the randomized control trial for detailed discussion and comparison. Eleven retrospective studies and one randomized control trial were meta-analyzed to compare existing evidence regarding the efficacy and clinical outcomes of vitrectomy with and without ILM peeling for idiopathic ERM.

## 2. Materials and methods

### 2.1 Literature search methods

The PRISMA checklist is described in S1 Table [22]. We selected relevant prospective or retrospective studies published before July 2016, by searching PubMed, Embase, Cochrane, Web of Science, Google Scholar, CNKI databases, FDA.gov, and ClinicalTrials.gov. The studies that compared the outcomes following vitrectomy, with and without ILM peeling, for treatment of idiopathic ERM disease were included. We did not apply language restrictions. We used relevant text words and medical subject headings that included all possible spellings of ERM and ILM peeling (detailed search strategy and records are shown in S2 Table). We considered all potentially eligible studies for review, irrespective of the primary outcome or language. Reference lists of all retrieved articles were searched manually to broaden the search. All scanned abstracts, studies, and citations were reviewed.

### 2.2 Criteria for inclusion, exclusion, and outcomes of interest

We included studies in this meta-analysis if they were randomized controlled trials or retrospective studies involving adults with idiopathic ERM, compared vitrectomy with and without ILM peeling, entailed pre- and postoperation monitoring of at least 3 months, and reported one change in the best corrected visual acuity (BCVA), central retinal thickness (CRT), or the rate of ERM recurrence at the end of the follow-up. The exclusion criteria were as follows: case series studies; studies with less than 3-month durations of follow up, studies including cases with a history of retinal detachment, proliferative diabetic retinopathy, retinal vascular occlusion disease that could lead to secondary macular pucker, and significant differences in the preoperative BCVA between the two groups. The outcomes assessed were extracted as follows: postoperative BCVA, rate of vision improvement, rate of ERM recurrence, and postoperative CRT at the end of follow-up, and change in CRT between baseline and the end of follow up.

### 2.3 Data extraction and quality assessment

Two investigators (WCC and CHL) independently reviewed study titles and abstracts, and studies that satisfied the inclusion criteria were retrieved for full-text assessment. Studies selected for detailed analysis and data extraction were analyzed by two investigators (WCC and CHL); disagreements were resolved by a third investigator (JHL). For each selected article, we extracted the following data: first author’s name, year of publication, ethnicity of the study population, participant numbers of both groups, and population characteristics including mean age, sex, follow-up duration, change in BCVA (mean [SD]), change in CRT (mean [SD]), and ERM recurrence rate at the end of the follow up. Because most of the selected studies were nonrandomized surgical studies, two independent reviewers (WCC and CHL) assessed the quality of eleven included retrospective trials according to the Newcastle–Ottawa Scale [23], and all the studies received scores >6 points. Only one enrolled randomized controlled trial was assessed according to the Cochrane Collaboration Reviewers’ Handbook for Systematic Reviews of Interventions [24] and revealed a low risk of bias.

### 2.4 Statistical analysis

We assessed the effect of vitrectomy with ILM peeling or not on five outcomes: data of functional efficacy, as assessed by postoperative BCVA and the rate of visual improvement; data of anatomical efficacy, as assessed by postoperative CRT and change of CRT; and the rate of ERM recurrence. We analyzed BCVA and CRT as continuous variables and reported mean difference (MD) with 95% CIs. For the analysis of the proportion of participants achieving VA improvement and those having recurrence of ERM at the end of follow up, we calculated risk ratios (RR) with 95% CIs. The I^2^ statistic, which was estimated using the DerSimonian–Laird method, was used to assess heterogeneity. An I^2^ value >50% indicated a moderate to high heterogeneity [25]. We presented pooled results from the fixed-effects and random-effects models to compare the differences based on the suggestions of a previous study [26]. If the treatment effect difference estimation on the basis of the fixed-effect and random-effect models were similar, we used the random-effects model to conclude because it can address interstudy heterogeneity, and is more conservative than the fixed-effect model. Egger’s regression and a funnel plot were used to test the symmetry of the pooled results [27].

For a high heterogeneity result, a meta-regression using an average summary value was used to determine the source of heterogeneity. Possible moderators (study type, quality score, age, and follow-up duration) were tested to explore heterogeneity. This study considered a *P* value of <0.05 to be significant for all analyses. Statistical analyses were conducted using the “metafor” [28] and “meta” [29] packages of R software, version 3.2.3.

## 3. Results

### 3.1 Study selection

**Fig 1:**
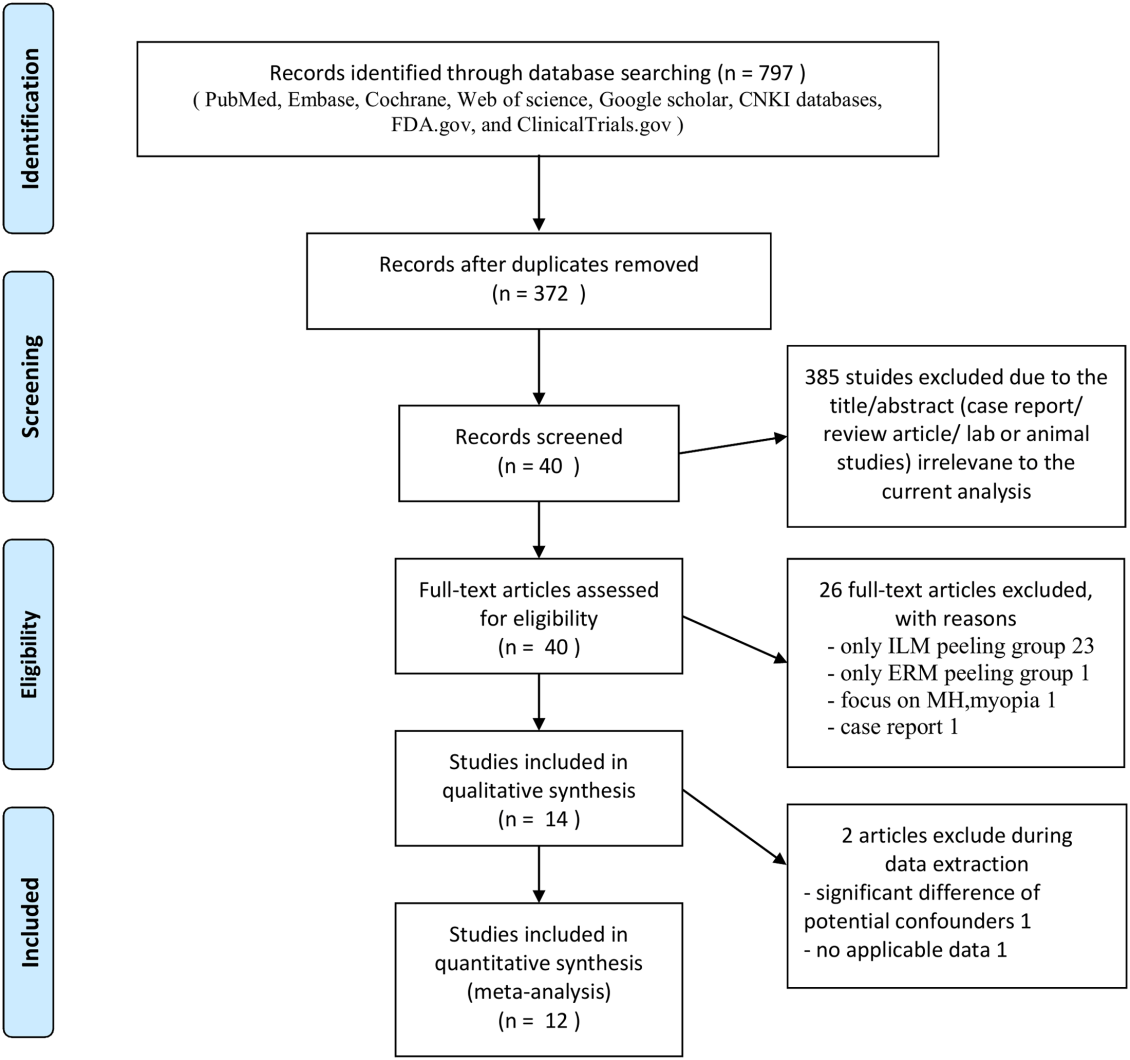
Flow diagram of the process of identifying eligible studies.

We identified 797 studies from PubMed, Embase, Cochrane, Web of Science, Google Scholar, CNKI databases, FDA.gov, and ClinicalTrials.gov, of which 12 (with data on 756 eyes) were included in our analysis. Fig 1 depicts the overall study identification process. For our analysis, 755 studies were unsuitable because they included duplicate studies, in vitro studies or animal studies, case reports, and review articles irrelevant to our topic. Forty records remained and 26 records were unrelated to the topic. Of the remaining 14 papers, 1 paper did not contain sufficiently detailed data for analysis and one had significant differences in potential confounders (preoperative BCVA). After screening all the titles, 11 retrospective studies and a randomized controlled trial (RCT) were included in our meta-analysis [20, 30–40]. A total of 12 retrospective studies were retrieved for more detailed evaluation; detailed data are presented in Tables 1 and 2.

**Table 1:** Summary of studies included in the meta-analysis.

Study	Country	Study type	Quality score		Group	No. of eyes	Mean age (yr)	Follow-up duration (mo)	Preoperative BCVA (LogMAR)	Preoperative CRT (um)
			>12	>6						
Kim et al., 2005	Korea	Retro	6	7	Non-ILM peeling	17	61.6	11.2	0.71+-0.50	418+-71
					ILM peeling	17	63.5	8.9	0.71+-0.16	483+-261
Kwok et al., 2005	Hong Kong	Retro	8	8	Non-ILM peeling	15	69.1+-8.3	47.9+-18.1	0.96+-0.18	-
					ILM peeling	20	63.8+-9.3	23.9+-5.5	0.77+-0.50	-
Liu et al., 2005	Japan	Retro	7	7	Non-ILM peeling	20	68.2+-6.9	3	0.44+-0.21	402.7+-110.3
					ILM peeling	18	69.4+-5.7	3	0.35+-0.26	385.6+-117.2
Mason et al., 2006	American	Retro	8	8	Non-ILM peeling	20	70(33–84)	16	-	-
					ILM peeling	20	68(53–78)	16	-	-
Lee et al., 2010	Korea	Retro	8	8	Non-ILM peeling	19	65.47+-7.66	18.2+-12.0	0.67+-0.34	398.42+-95.43
					ILM peeling	21	63.43+-7.18	18.05+-11.81	0.68+-0.21	409.43+-111.62
Pournaras et al., 2011	Switzerland	Retro	7	8	Non-ILM peeling	15	77.1+-6.7	41.9+-35.6	0.48+-0.22	-
					ILM peeling	24	73.3+-10.6	24.0+-12.6	0.58+-0.40	401+-96
Chuang et al., 2012	Taiwan	Retro	8	8	Non-ILM peeling	61	62.08+-10.52	21.97+-11.08	0.14+-0.11	462.70+-83.90
					ILM peeling	20 (TA) 23 (ICG)	63.80+-9.63 63.26+-9.72	17.55+-4.19 20.26+-9.51	0.21+-0.18 0.14+-0.10	470.30+-87.34 467.43+-94.56
Oh et al., 2013	Korea	Retro	7	8	Non-ILM peeling	23	64	12	0.35+-0.16	-
					ILM peeling	20	65.3	12	0.44+-0.21	-
Ahn et al., 2014	Korea	Retro	7	8	Non-ILM peeling	69	63.9+-11.1	12	0.38+-0.19	456+-77.4
					ILM peeling	40	64.3+-10.0	12	0.31+-0.21	445+-99.3
Reilly et al., 2015	American	Retro	6	7	Non-ILM peeling	78	-	-	0.324(phakic) 0.284(pseudophakic)	-
					ILM peeling	51	-	-	0.292(phakic) 0.316(pseudophakic)	-
Jung et al., 2016	American	Retro	8	8	Non-ILM peeling	43	68.6	36.3	0.53	-
					ILM peeling	42	71.5	29.9	0.52	-
Ripandell et al., 2015	Italy	RCT			Non-ILM peeling	30	-	12	0.298+-0.1082	473.80+-75.70
					ILM peeling	30	-	12	0.306+-0.214	464.20+-89.20

Retro: retrospective study; Quality score: assessed the quality of retrospective studies according to the Newcastle–Ottawa Scale (NOS), we formed two groups by using different scoring methods assuming that the follow-up time was sufficiently long, 6 months or 12 months; yr: year; mo: month

**Table 2:** Summary of studies included in the meta-analysis.

Study	Group	Postoperative BCVA (LogMAR)	VA improvement (n/total)	Postoperative CRT(um)	CRT decrease (um)	ERM recurrence rate % (n/total)
Kim et al., 2005	Non-ILM peeling	0.37+-0.22	9/17 (>2lines)	282+-72	197+-152	0% (0/17)
	ILM peeling	0.54+-0.22	8/17 (>2lines)	328+-55	125+-138	0% (0/17)
Kwok et al., 2005	Non-ILM peeling	0.65+-0.32	12/15 (>2lines)	-	-	20% (3/15)
	ILM peeling	0.46+-0.37	11/20 (>2lines)	-	-	10% (2/20)
Liu et al., 2005	Non-ILM peeling	0.19+-0.17	12/20 (>2lines)	295.2+-81.6	-	-
	ILM peeling	0.20+-0.19	8/18 (>2lines)	307.2+-60.8	-	-
Mason et al., 2006	Non-ILM peeling	-	100% (no mention)	-	-	20% (4/20)
	ILM peeling	-	70% (no mention)	-	-	0% (0/19)
Lee et al., 2010	Non-ILM peeling	0.32+-0.23	-	282.53+-95.71	115.89+-107.48	0% (0/19)
	ILM peeling	0.20+-0.17	-	335.24+-76.91	74.19+-79.33	0% (0/21)
Pournaras et al., 2011	Non-ILM peeling	0.37+-0.42	8/15 (no mention)	268+-98	-	-
	ILM peeling	0.32+-0.39	19/24 (no mention)	307+-49	-	-
Chuang et al., 2012	Non-ILM peeling	0.41+-0.55	-	299.44+-63.57	-	13% (8/61)
	ILM peeling	0.39+-0.57 0.31+-0.45	-	295.35+-86.82 301.74+-74.04	-	0% (0/43)
Oh et al., 2013	Non-ILM peeling	0.40+-0.18(3m) 0.43+-0.24(6m) 0.50+-0.28(12m)		-	-	-
	ILM peeling	0.56+-0.26(3m) 0.46+-0.26(6m) 0.54+-0.28(12m)		-	-	-
Ahn et al., 2014	Non-ILM peeling	0.11+-0.12	-	356+-58.9	-	20.3% (14/69)
	ILM peeling	0.17+-0.17	-	342+-38.9	-	7.5% (3/40)
Reilly et al., 2015	Non-ILM peeling	0.226(12m) 0.206(12m)	-	-	-	5.1% (4/78)
	ILM peeling	0.113(12m) 0.214(12m)	-	-	-	3.9% (2/51)
Jung et al., 2016	Non-ILM peeling	0.41(3m) 0.35(6m) 0.38(12m) 0.33(24m) 0.32(36m)	-	-	95.7 - 130.2+-108.5 136.5+-116.1 136.9+-110.5	13% (5/39)
	ILM peeling	0.38(3m) 0.27(6m) 0.33(12m) 0.18(24m) 0.23(36m)	-	-	67.4 - 86.2+-90.0 105+-90.1 84.1+-90.2	0% (0/42)
Ripandell et al., 2015	Non-ILM peeling	0.034+-0.1082	-	351.03+-40.24	-	0% (0/30)
	ILM peeling	0.048+-0.0822	-	376.9+-45.12	-	0% (0/30)

BCVA: best-corrected visual acuity; CRT: central retinal thickness

### 3.2 Study characteristics

The 12 studies were published between 2005 and 2016 (one was published in 2016). In total, 756 eyes, comprising 410 and 346 eyes without and with ILM peeling, respectively, that underwent treatment of idiopathic macular pucker were included in this meta-analysis. Of the included studies, 11 studies were retrospective, and 1 was an RCT; and seven, two, and three studies were conducted in Asia, Europe, and USA, respectively. The sample size of each study varied from 34 to 139 eyes. The baseline characteristics of each included study, such as duration of follow up, preoperative BCVA, and preoperative CRT are listed in Table 1. Table 2 also presents postoperative BCVA, postoperative CRT, change in CRT reduction, vision improvement rate (which is defined by an improvement in VA of ≥2 Snellen lines in three studies), and ERM recurrence rates (which are defined as any evidence of a recurrent macular ERM on spectral domain optical coherence tomography). Five studies mentioning postoperative complications, and retinal detachment, vitreous hemorrhage, punctate retinal hemorrhage, cataract, and retinal tear are listed in Table 3. Of these 12 studies, seven used ICG, one used triamcinolone and ICG, and two used the BBG assistant for ILM staining. Two studies did not mention the type of stain in the context. Phakia and pseudophakia were also noted in some studies. The 11 retrospective studies contained the confounding factor of lens status without strict inclusion criteria for prior lens variability; however, the RCT did have a strict inclusion criteria for prior lens variability.

**Table 3:** Surgery-related features of studies included in the meta-analysis.

Study	Group	Complication	Stain	Vitrectomy	Phakia/IOL	
					Pre op	Post op
Kim et al., 2005	Non-ILM peeling	No mention		-	15/2	-
	ILM peeling		ICG		12/5	-
Kwok et al., 2005	Non-ILM peeling	1 post op RD		-	14/1	-
	ILM peeling	1 post op RD	ICG		17/3	-
Liu et al., 2005	Non-ILM peeling	No mention		-	-	1/19
	ILM peeling		-		-	0/18
Mason et al., 2006	Non-ILM peeling	10% vitrous hemorrhage, no infection, no RD, no phototoxic, no RPE damage		25G forceps	-	-
	ILM peeling		ICG		-	-
Lee et al., 2010	Non-ILM peeling	No significant complication observed		-	18/1	2/17
	ILM peeling		ICG		19/2	1/20
Pournaras et al., 2011	Non-ILM peeling	No significant intraoperative or postoperative complications observed		20G	0/15	0/15
	ILM peeling		ICG		0/24	0/24
Chuang et al., 2012	Non-ILM peeling	No mention		-	-	-
	ILM peeling		TA/ICG		-	-
Oh et al., 2013	Non-ILM peeling	5 cataract(21.7%), 14 punctate retinal hemorrhage(60.9%) 6 cataract(30.0%), 13 punctate retinal hemorrhage(65.0%) 1 vitreous hemorrhage(5.0%)		20G	18/5	17/6
	ILM peeling		ICG	-	16/4	16/4
Ahn et al., 2014	Non-ILM peeling	No mention		23G	99/10	33/36
	ILM peeling	6 cataract(30.0%), 13 punctate retinal hemorrhage(65.0%) 1 vitreous hemorrhage(5.0%)	ICG			12/28
Reilly et al, 2015	Non-ILM peeling	1 RD with PVR at post op 5 weeks, 1 choroidal neovascular membrane, 1 retinal tear s/p laser retinopexy		-	66/63	32/46
	ILM peeling		-			20/31
Jung et al., 2016	Non-ILM peeling	No mention		23G	-	-
	ILM peeling		BBG		-	-
Ripandell et al., 2015	Non-ILM peeling	No adverse events		23G	0/30	0/30
	ILM peeling		BBG		0/30	0/30

IOL: intraocular lens; Preop: preoperation; Postop: postoperation; RD: retinal detachment; RPE: retinal pigment epithelium; PVR: proliferative vitreoretinopathy; ICG: indocyanine green; TA: triamcinolone acetonide; BBG: Brilliant Blue G

### 3.3 Efficacy analysis

**Fig 2:**
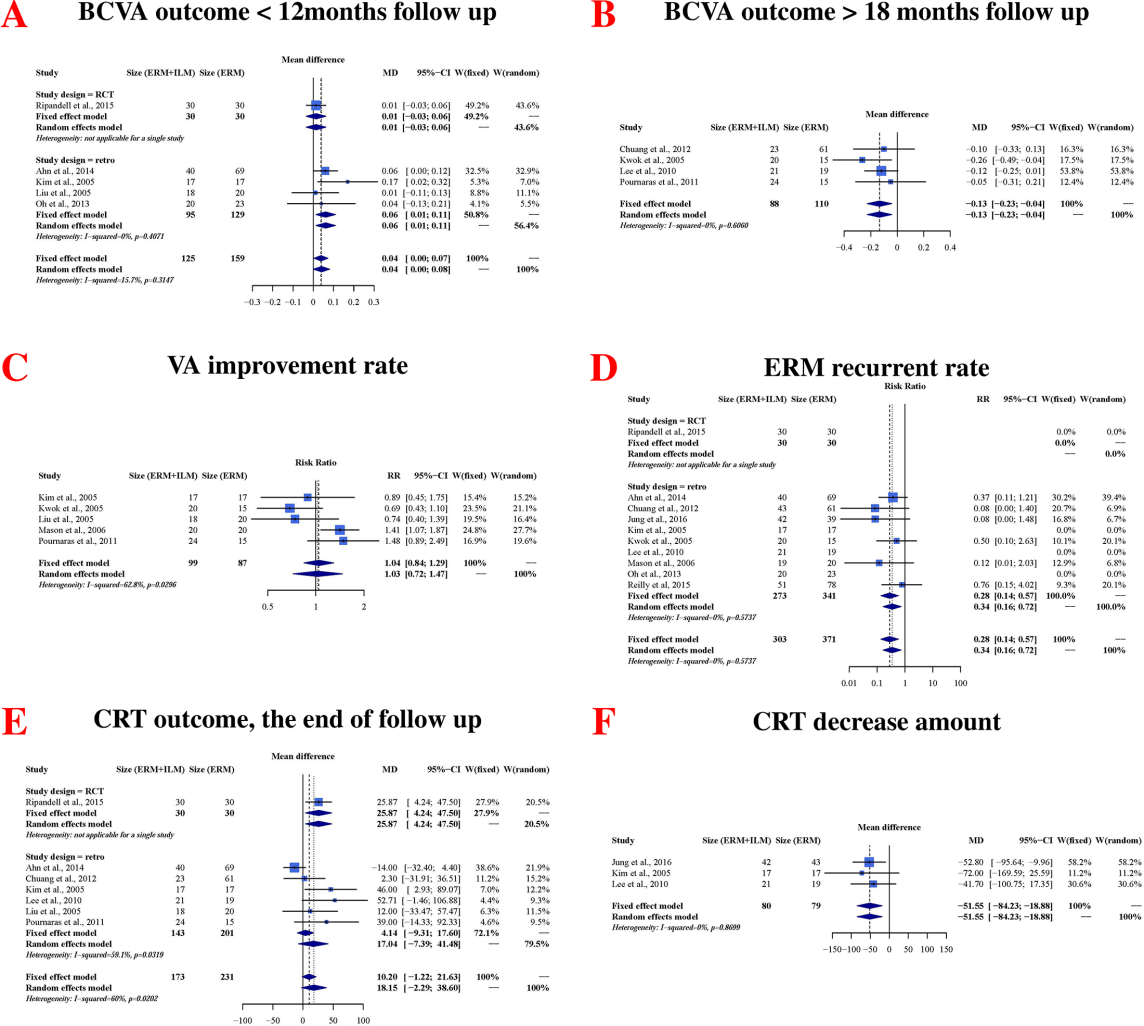
Main results from the meta-analysis of vitrectomy with epiretinal membrane (ERM) and ILM peeling or with only ERM peeling in idiopathic ERM. (A) Best-corrected visual acuity (BCVA) <12 months follow-up duration after surgery; vitrectomy with only ERM peeling yielded significantly better results; (B) BCVA >18 months follow-up duration after surgery; vitrectomy with ERM+ILM peeling yielded significantly better results; (C) rate of improvement in visual acuity, defined as ≥2 Snellen lines at the end of follow-up; the difference between the two groups was nonsignificant (D) ERM recurrence rate; was significantly lower in the ERM + ILM peeling group than in the ERM peeling only group (E) central retinal thickness (CRT) at the end of follow-up; the difference between the two groups was nonsignificant; (F) CRT reduction at the end of follow-up; CRT reduction was significantly higher in the ERM peeling group than in the ERM+ ILM peeling group.

Fig 2 depicts the six main outcomes from our meta-analysis. In a pooled analysis of nine trials involving 482 eyes, five trials indicated that the non-ILM peeling group exhibited better BCVA in the <12-month follow-up period after surgery than did the ILM peeling group (mean difference [MD] = 0.04, 95% CI: 0.0007 to 0.008; *P* = 0.0460), and no significant heterogeneity was observed between the two groups (heterogeneity *P* = 0.3147, I^2^ = 15.7%) (Fig 2). Although the RCT only revealed that the differences were not significant, the combined retrospective studies with RCT rendered the differences significant.

In the other four trials, the ILM peeling group revealed better BCVA in a follow-up time >18 months greater than that of the non-ILM peeling group (MD = −0.13, 95% CI: −0.23 to −0.04; *P* = 0.0049), with no significant heterogeneity between studies (heterogeneity *P* = 0.6060, I^2^ = 0%; Fig 2).

In the evaluation of the rate of VA improvement after surgery, the pooled data of five studies including 186 eyes showed no significant difference between the two groups (RR = 1.03, 95% CI:0.72 to 1.47; *P* = 0.8802). The heterogeneity between studies was statistically significantly moderate (heterogeneity *P* = 0.0296, I^2^ = 62.8%; Fig 2).

The postoperative CRT was assessed in seven studies, involving a total of 404 eyes. Pooling the data of these studies showed no significant differences between the ILM peeling and non-ILM peeling groups (MD = 18.15, 95% CI: −2.29 to 38.60; *P* = 0.0818). The heterogeneity between studies in this analysis was statistically significant (heterogeneity *P* = 0.0202, I^2^ = 60%; Fig 2).

We assessed the change in CRT after operation in three studies, involving a total of 159 eyes. Pooling the data from these studies showed that the non-ILM peeling group had a higher mean reduction in CRT than did the ILM peeling group (MD = −51.55, 95% CI: −84.23 to −18.88; *P* = 0.0020), with no statistically significant heterogeneity between studies (heterogeneity *P* = 0.8699, I^2^ = 0%; Fig 2).

The rate of ERM recurrence after surgery was reported in 10 studies involving a total of 674 eyes. By using a random-effects model, the rate of ERM recurrence after initial surgery was revealed to be higher in the non-ILM peeling group than that in the ILM peeling group (RR = 0.34, 95% CI: 0.16 to 0.72; *P* = 0.0048; Fig 2). The heterogeneity between the studies was not statistically significant (heterogeneity *P* = 0.5737, I^2^ = 0%).

Funnel plot and Egger’s regression were used to determine the potential bias of pooled results. In all six funnel plots, we found no evidence of asymmetry from a visual observation and Egger’s regression yielded the same result (*P* value of Egger’s regression > 0.05; S1 Fig). Egger’s regression test indicated no evidence of publication bias among the included studies or pooled results in this meta-analysis. A summary of all the results is presented in Table 4.

**Table 4:** Summary of results.

**Continuous variable results**	**RCT**		**Retrospective**			**Combine**			**Egger’s test**
**Random effects model**	**MD (95% CI)**	**p value**	**MD (95% CI)**	**p value**	**I**^**2**^	**MD (95% CI)**	**p value**	**I**^**2**^	
BCVA outcome < 12months follow up	0.01(-0.03–0.06)	0.5725	0.06(0.01–0.11)	0.0122	0%	0.04(0.00–0.08)	0.0460	15.7%	0.408
BCVA outcome > 18 months follow up^a^	-		-0.13(-0.23–0.04)	0.0049	0%	-	-	-	0.943
CRT outcome, the end of follow up	25.87(4.24–47.50)	0.0191	17.04(-7.39–41.48)	0.1717	59.1%	18.15(-2.29–38.60)	0.0818	60%	0.109
CRT reduction amount^a^	-		-51.55(-84.23–18.88)	0.0020	0%	-	-	-	0.661
**Category variable results**	**RCT**		**Retrospective**			**Combine**			**Egger's test**
**Random effects model**	**RR (95% CI)**	**p value**	**RR (95% CI)**	**p value**	**I**^**2**^	**RR (95% CI)**	**p value**	**I**^**2**^	
VA improvement rate^a^	-	-	1.03(0.72–1.47)	0.8802	62.8%	-	-	-	0.239
ERM recurrence rate	-^b^	-^b^	0.34(0.16–0.72)	0.0048	0%	0.34(0.16–0.72)	0.0048	0%	0.083

*P* values: all represent the random effects model result

I^2^: index for assessing heterogeneity; value >50% indicates a moderate to high heterogeneity

Egger’s test: *P* value of Egger’s regression for asymmetry assessment

^a:^ The randomized controlled trial (RCT) did not provide data of BCVA outcomes >18 months follow-up, VA improvement rate, and CRT reduction amount; hence, combining the RCT with the analysis of retrospective studies in these three outcomes was not applicable.

^b:^ The RCT revealed no ERM recurrence in total 60 cases.

Because high heterogeneities were found in the rate of VA improvement and postoperative CRT, we further analyzed the sources of heterogeneities using meta-regression. Table 5 lists the moderator effects from study type, quality score, age, and follow-up duration. However, all of the potential moderators showed no significant effects on heterogeneity in the meta-regression analysis.

**Table 5:** Meta-regression analysis of heterogeneity and moderator effects of study characteristics on high heterogeneity outcomes.

	VA improvement rate			CRT outcome, the end of follow up		
	n	RR (95% CI)	p value	n	Slope (95% CI)	p value
Study type (retrospective is ref.)^a^	NA	NA	NA	7	8.828 (-46.220 to 63.877)	0.7533
Quality score	5	1.421 (0.683 to 2.958)	0.3478	6	-18.431 (-69.977 to 33.114)	0.4834
Mean age	5	1.060 (0.977 to 1.150)	0.1591	6	1.511 (-4.805 to 7.827)	0.6392
Study duration	5	1.003 (0.970 to 1.038)	0.8506	7	0.673 (-2.243 to 3.588)	0.6510

^a:^ the randomized controlled trial did not provide data on VA improvement rate; hence, meta-regression analysis of study type was not applicable.

## 4. Discussion

Our results revealed that—compared with vitrectomy with ILM peeling—vitrectomy without ILM peeling achieved better BCVA in a follow-up duration shorter than 12 months. By contrast, better BCVA was observed in the ILM peeling group in studies in which the follow-up duration was longer than 18 months. Furthermore, non-ILM peeling group exhibited higher reduction in postoperation CRT and a higher rate of ERM recurrence than did the ILM peeling group. These data thus support that vitrectomy with ILM peeling is a preferable surgical treatment that can improve long-term postoperative BCVA of idiopathic macular pucker and lower the recurrence rate of ERM.

In the management of idiopathic macular pucker, pars planar posterior vitrectomy with ERM peeling and ILM peeling have been reported to successfully remove macular pucker, improve VA, and relieve metamorphopsia [41]. However, the safety and efficacy of ILM peeling remain controversial. Some studies have mentioned that ILM peeling may not result in improved postoperative BCVA compared with only peeling the ERM, and have reported complications, including the formation of macular holes [42]. To date, only one meta-analysis has discussed the postoperative BCVA, vision improvement, and ERM recurrence rates with limited data and unspecified conclusions [21]. This meta-analysis showed no significant difference in ERM recurrence rates between the ILM peeling and non-ILM peeling groups. Furthermore, some anatomical damage after ILM peeling including dissociation of the nerve fiber layer and inner retinal dimpling was reported by recent studies [43–45]. The functional significance of the anatomical damage is still controversial [37, 43, 46, 47]. In this context, we performed a more complete meta-analysis to compare the postoperative BCVA, vision improvement rates, ERM recurrence rates, postoperative CRT, and CRT reduction between the two groups by including more retrospective studies and RCTs, and providing more powerful evidence in the visual and recurrence outcomes discussion.

**Fig 3:**
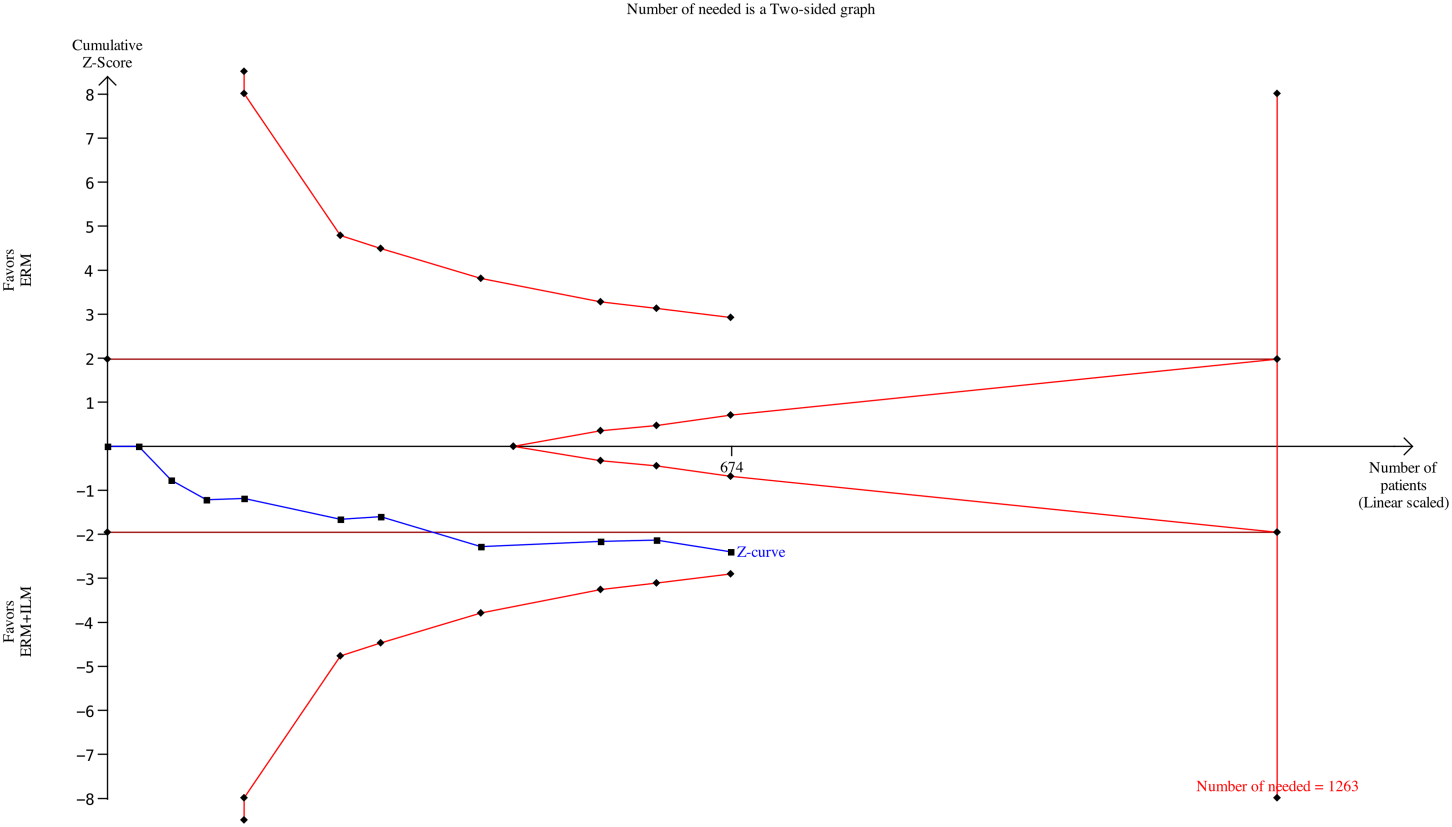
Trial Sequential Analysis (TSA) of the ERM recurrence rate outcome in this meta-analysis. TSA is a methodology that includes a sample size calculation for a meta-analysis with the threshold of statistical significance. The detailed settings of this TSA were shown as follows: Significance level = 0.05; Power = 0.80; incidence of control = 2.3; relative risk reduction = 30%; I^2^ = 0%. Finally, the number of required samples is 1263 but this meta-analysis collected only 674 samples.

Our meta-analysis has highlighted significant findings that support the benefits of ILM peeling for the treatment of idiopathic ERM with a lower recurrence rates in the ILM peeling group than in the non-ILM peeling. De Bustros et al. promoted a hypothesis regarding ILM as a growth bridge for proliferation of fibroblasts, glial cells, and astrocytes from the retina to form the ERM [3]. Some studies have reported the benefits of ILM peeling in the ERM surgery in reducing the risk of ERM recurrence. Our trial is the first meta-analysis to present evidence to indicate that the ERM recurrence rate is related to ILM peeling. Compared with the single randomized controlled trial [20], no recurrence in both the groups was observed at the end of 12-month follow-up. Ripandelli et al. mentioned that the result may be related to the limited follow-up time, and that a longer observation period is necessary. We enrolled 10 studies investigating ERM recurrence with a follow-up time range from 8.9 to 47.9 months, which is longer than the follow up time of RCT. A Trial Sequential Analysis (TSA) was performed for calculating the required sample size for this factor. The number of necessary samples is 1263; we collected 674 samples in this meta-analysis. Although this number was insufficient, Fig 3 illustrates that the cumulative z-curve crosses the traditional boundary near the trial sequential monitoring boundary and tends to reach the required information size. We can fairly suppose that if we had more samples for this factor, the results would be consistent with our findings and exhibit strong validity. Our results have a crucial role in the discussion of ERM recurrence with or without ILM peeling. However, regarding the study design and methodology of our enrolled studies, the definition of the ERM recurrence differed among the studies. More highly qualified RCTs are necessary to provide robust evidence to prove our hypothesis.

Another significant finding from our meta-analysis is a more efficient effect on the reduction of CRT in the non-ILM peeling group than in the ILM peeling group. This effect was also noted in the RCT conducted by Ripandelli et al, in which faster reduction of foveal thickness and higher central foveal cube volume were observed in the non-ILM peeling group than in the ILM peeling group [20]. Moreover, this higher reduction in CRT was not achieved along with a significantly thinner postoperative CRT, better BCVA, or pronounced higher improvement rate in the VA between both the groups. These findings were consistent with those of Ripandelli et al. These findings were also reported by Chang et al. They mentioned a higher proportional decrease in central macular thickness in the single-peeling group than in the double-peeling group [16]. Chang et al. considered that it may be related to an increased disruption or swelling of the inner retinal layers postoperatively in the double-peeling group. Wollensak et al. also demonstrated that ILM may be the structure that contributes to the biomechanical strength of the retina [48], thereby explaining the ILM maintaining the retinal tension. Despite the residual ILM function, we thought that the longer operation time and more complex peeling procedure in the double-peeling group leading to more turbulent retinal tissue may contribute to the ILM peeling group exhibiting an decreased reduction in CRT change in comparison with the non-ILM peeling group (with the effect of potential retinal edematous changes due to increased damage). Longer operation time may cause more potentially phototoxicity but we did not find enough evidence to prove the association between light damage and CRT change currently [49–51]. Moreover, the postoperative CRTs at the end of follow-up were not significantly different between the two groups. The high heterogeneity between studies in this analysis reflected the differences in study design. The different CRT definition and measurement by different OCT machine with different protocol exist in each studies. TSA indicated the lack of numbers of required samples which ensure that the results are credible and significant. Although the RCT showed significant CRT thinning in the non-ILM peeling group, we still believed that increased and larger RCTs are necessary to verify whether the postoperative CRT outcome or CRT reduction were affected by ILM peeling.

In our meta-analysis, the postoperative BCVA significantly better within the <12 months follow-up time in the non-ILM peeling group, but showed converse results in the longer follow-up >18 months in the ILM peeling group. This result was consistent with that of Liu et al [21]. We maintained a conservative attitude toward these results because of the marked influence from confounding factors such as post-vitrectomy cataract formation, variations in the VA measurement method in each trial, and the conversion of different units to logMAR. Among our enrolled studies, only the RCT adopt Early Treatment Diabetic Retinopathy Study (ETDRS) chart to evaluate VA in comparison of other retrospective studies using Snellen chart, which may lead to some inconsistency for the BCVA outcomes. The cases included in the Ripandelli et al. had the visual acuity better than 20/200 which is showed less pronounced difference between ETDRS and Snellen chart [52]. Anatomical and functional changes in the photoreceptor cells postoperation, particularly in the ILM peeling method, were estimated by microperimetry in many trials. These factors can potentially negatively affect the improvement of BCVA [20, 34, 39, 40, 53–60]. In the RCT, Ripandelli et al. demonstrated no difference in BCVA between the groups until 12 months after ERM surgery and provided more substantial evidence of BCVA change at 12 months follow-up [20]. Thus, we cannot draw conclusions regarding the postoperative BCVA between these two different surgery methods in the current limited evidence-based context. More precise and controlled confounding factors and longer RCTs are necessary to confirm the visual outcomes post vitrectomy with and without ILM peeling surgery for the treatment of idiopathic ERM disease. Furthermore, our meta-analysis revealed that VA improvement rates were not significantly different between the two groups. The improvement of BCVA in patients undergoing vitrectomy with and without ILM peeling were similar. We performed TSA and determined that even if more case numbers are enrolled in the trials, the result remains nonsignificantly different between the with and without ILM groups. The lack of differences in VA improvement between the groups is likely related to the broad definition of ≥2 Snellen lines in each trial. This may have resulted from variations in the VA improving range in each case. This outcome resulted in no comparison of the surgical efficacy between the two groups. We can only consider vitrectomy with and without ILM peeling as two beneficial surgical methods for treating patients with idiopathic ERM that result in postoperative VA outcome improvement.

Several limitations of the present meta-analysis should be acknowledged. First, most of the studies available for this meta-analysis were retrospective studies; hence, evident selection bias and observer bias with regard to the adoption of the operative approach are possible. Second, as is known, we relied on the tabulated data for the meta-analysis, rather than on individual patient data. However, meta-analyses have increased power compared with individual studies, and provide more accurate confidence intervals. Third, all the enrolled trials did not prove the use of single-peeling or double-peeling procedures with pathological tissue proof. Some errors in ERM peeling or ILM peeling depend on individual experiences. Gaudric et al. have histologically compared functional and anatomical results between single-and double-peeling groups [15]. It may resolve the variations in peeling techniques among different surgeons. However, the problem of intersurgeon variability, including surgical duration and peeling technique, and different vitrectomy gauge usage were difficult to solve despite being commonly encountered. The difference of the microincision vitrectomy surgery (MIVS) had been reported faster visual recovery and less postoperative inflammation in smaller gauge MIVS observed in days to weeks follow-up duration [61–63]. However, our visual outcomes were long term result rather than short term recovery. Our BCVA outcomes and VA improvement rate may not be interfered with the different MIVS procedures bias. Fourth, the confounding factor, postoperative cataract formation, was not eliminated in most trials in our meta-analysis. Different ratios of preoperative patients with phakia and aphakia were observed among different trials. Patients with phakia may exhibit lens opacity, thereby influencing VA following ERM surgery. Additional RCTs with strict inclusion criteria should be conducted to support the evidence in discussion of post BCVA in ERM surgery. Furthermore, the method of using ICG stain assistance for ILM peeling varied in each trial. To date, the toxicity of different stain durations and osmolality toward retinal tissue remains controversial and uncertain [64–66]. Some reports revealed ICG might lead to optic nerve atrophy in the long-term and persistent visual field defects [67–69]. We supposed that some effects of differences in stain usage on the outcomes of BCVA of each trial are possible. However, it still lacked of strong evidence to support the association between ICG toxicity and the expression of BCVA or CRT outcomes. Additionally, the different measurement of the visual acuity, the different definition of the ERM recurrence and the different OCT machine with different protocol measuring CRT may all cause the bias. Finally, the adequate duration of follow-up time to reveal the effect of operation was unknown. Our meta-analysis included follow-up times from at least 3 months up to 41.9 months. Although in the meta-regression, our analysis of the trials in the meta-analysis resulted in nonsignificant influences on the six main outcomes on the follow-up duration, we believe that higher numbers of follow-up trials of a longer duration are required to reveal the actual outcomes; this applies particularly to the recurrence rate and CRT because iatrogenic effects, such as residual ERM and postoperative retinal edema, influence the outcome of surgery.

In conclusion, our meta-analysis provides important evidence for exploring vitrectomy with or without ILM peeling in idiopathic ERM treatment. Vitrectomy with ILM peeling might provide higher VA improvement after long-term follow up and might reveal poorer results in CRT reduction than does vitrectomy without ILM peeling. ERM recurrence rate is significantly lower after vitrectomy with ILM peeling than after vitrectomy without ILM peeling according to currently limited data. Further studies are necessary to establish the optimal visual and anatomical outcome of this surgery. Large, prospective randomized clinical trials are necessary to draw valid results and confirm our current conclusion.

## Supporting information

S1 Table PRISMA 2009 checklist. (DOC)

S2 Table Search strategies and detailed records. (DOCX)

S1 Fig Funnel plots of the six main outcomes. (PDF)
