# Supplementary material for: Vitrectomy with or without internal limiting membrane peeling for idiopathic epiretinal membrane: A meta-analysis
Source: PLoS One. 2017 Jun 16;12(6):e0179105. doi: 10.1371/journal.pone.0179105 (PMC5473547; doi:10.1371/journal.pone.0179105)
Supplement: S2 Table — (DOCX) [file pone.0179105.s002.docx]

**S2 Table. Search strategies and detailed records.**

| **Relevant text of ILM peeling**   1. Internal limiting membrane peeling 2. Vitrectomy with internal limiting membrane peeling 3. Pars plana vitrectomy with internal limiting membrane peeling 4. Vitrectomy with internal limiting membrane peel 5. Pars plana vitrectomy with internal limiting membrane peel 6. Internal limiting membrane peel 7. 1 or 2 or 3 or 4 or 5 or 6 | **Relevant text of ERM**   1. Macular pucker 2. Epiretinal membrane 3. Idiopathic epiretinal membrane 4. Epimacular membrane 5. Idiopathic epimacular membrane 6. Retinal membrane 7. Idiopathic retinal membrane 8. Macular membrane 9. Idiopathic macular membrane 10. 8 or 9 or 10 or 11 or 12 or 13 or 14 or 15 or 16   **Combined (Final strategy)**   1. 7 and 17 |
| --- | --- |

Web sites and uniform resource locator:

**PubMed**: <http://www.ncbi.nlm.nih.gov/pubmed>

**Embase**: https://www.embase.com

**Cochrane:** http://www.cochranelibrary.com

**Web of science**: http://www.webofknowledge.com

**Google scholar:** https://scholar.google.com.tw

**CNKI databases:** http://oversea.cnki.net/kns55/default.aspx

**FDA.gov**: http://www.fda.gov

**ClinicalTrials.gov.**: https://clinicaltrials.gov
