# Supplementary figures and images for: Vitrectomy with or without internal limiting membrane peeling for idiopathic epiretinal membrane: A meta-analysis
Source: PLoS One. 2017 Jun 16;12(6):e0179105. doi: 10.1371/journal.pone.0179105 (PMC5473547; doi:10.1371/journal.pone.0179105)

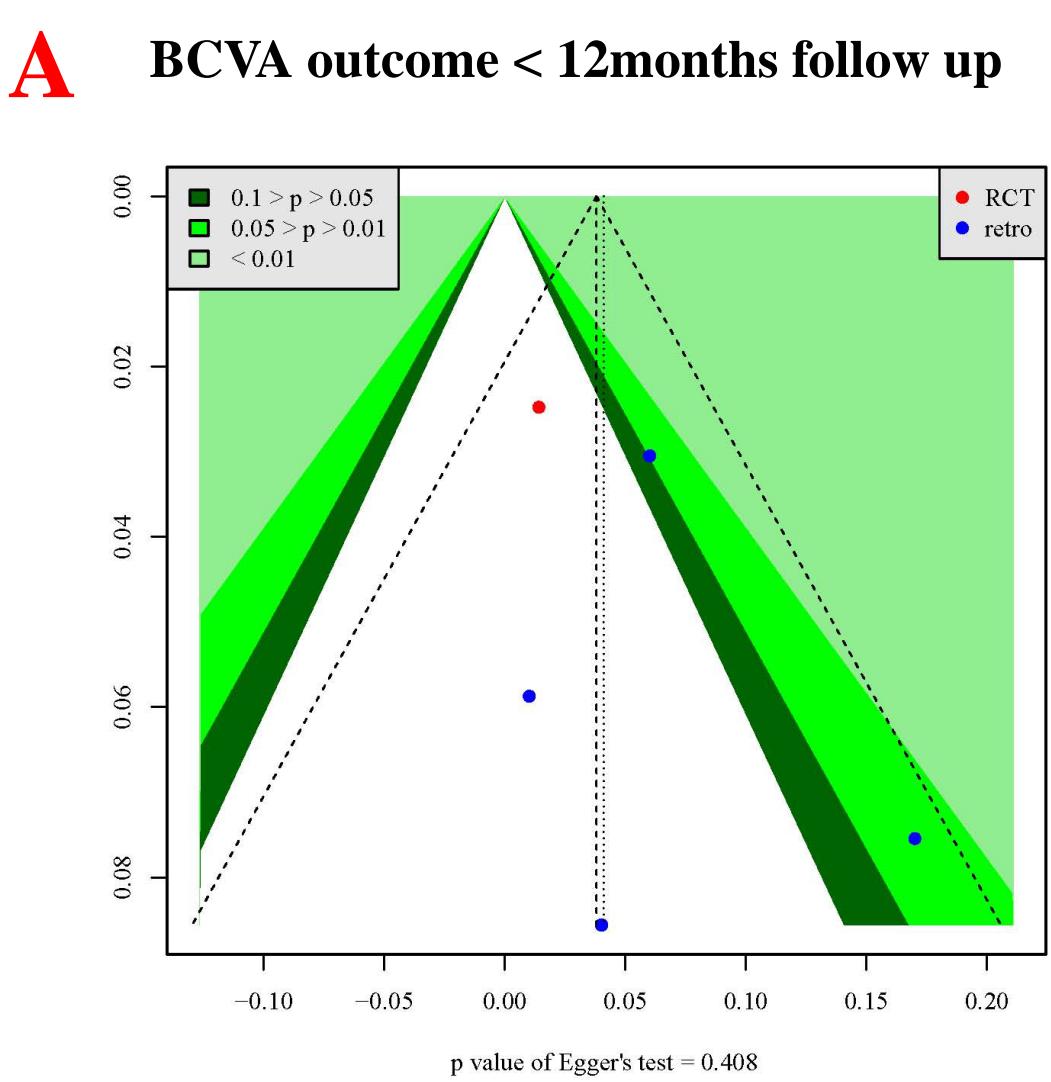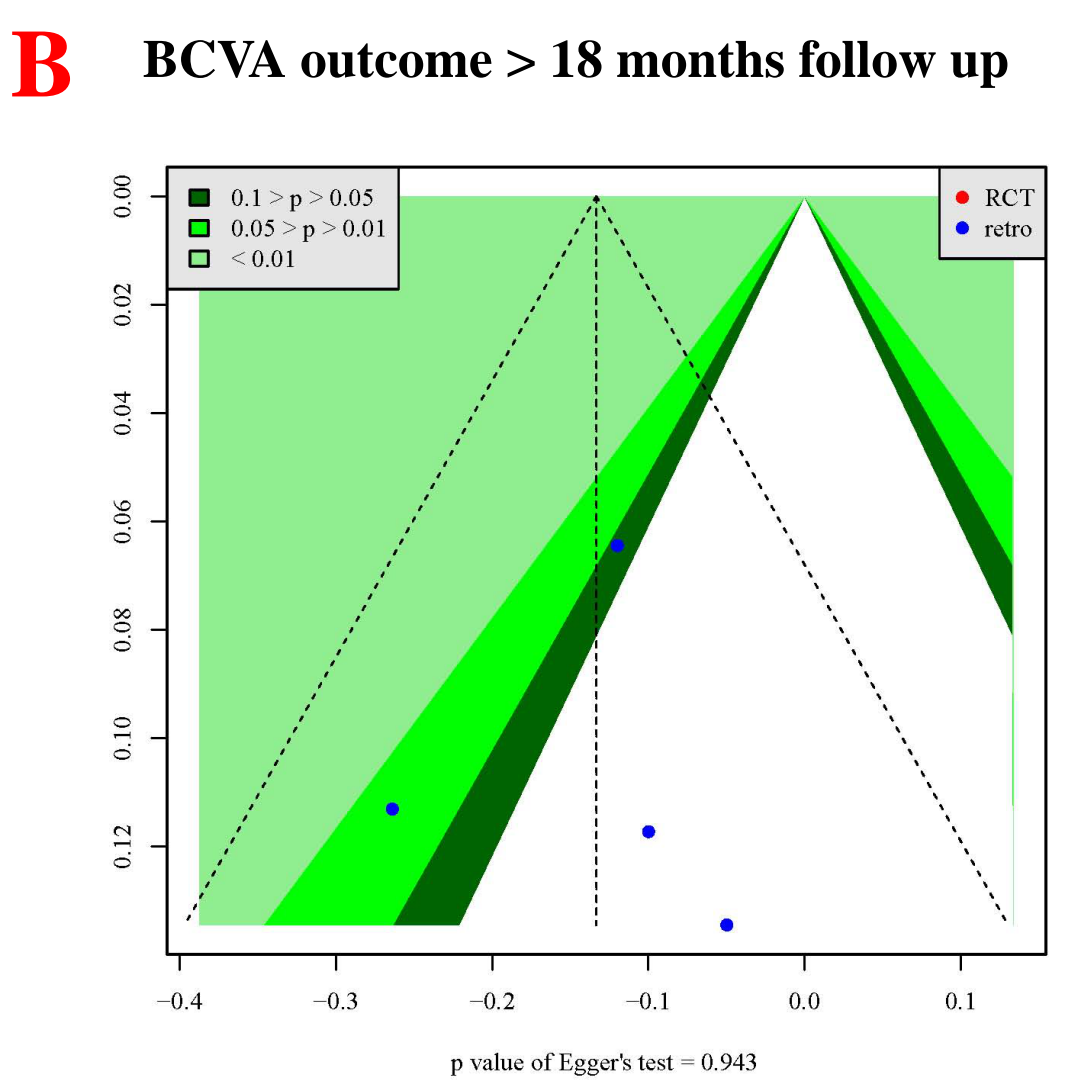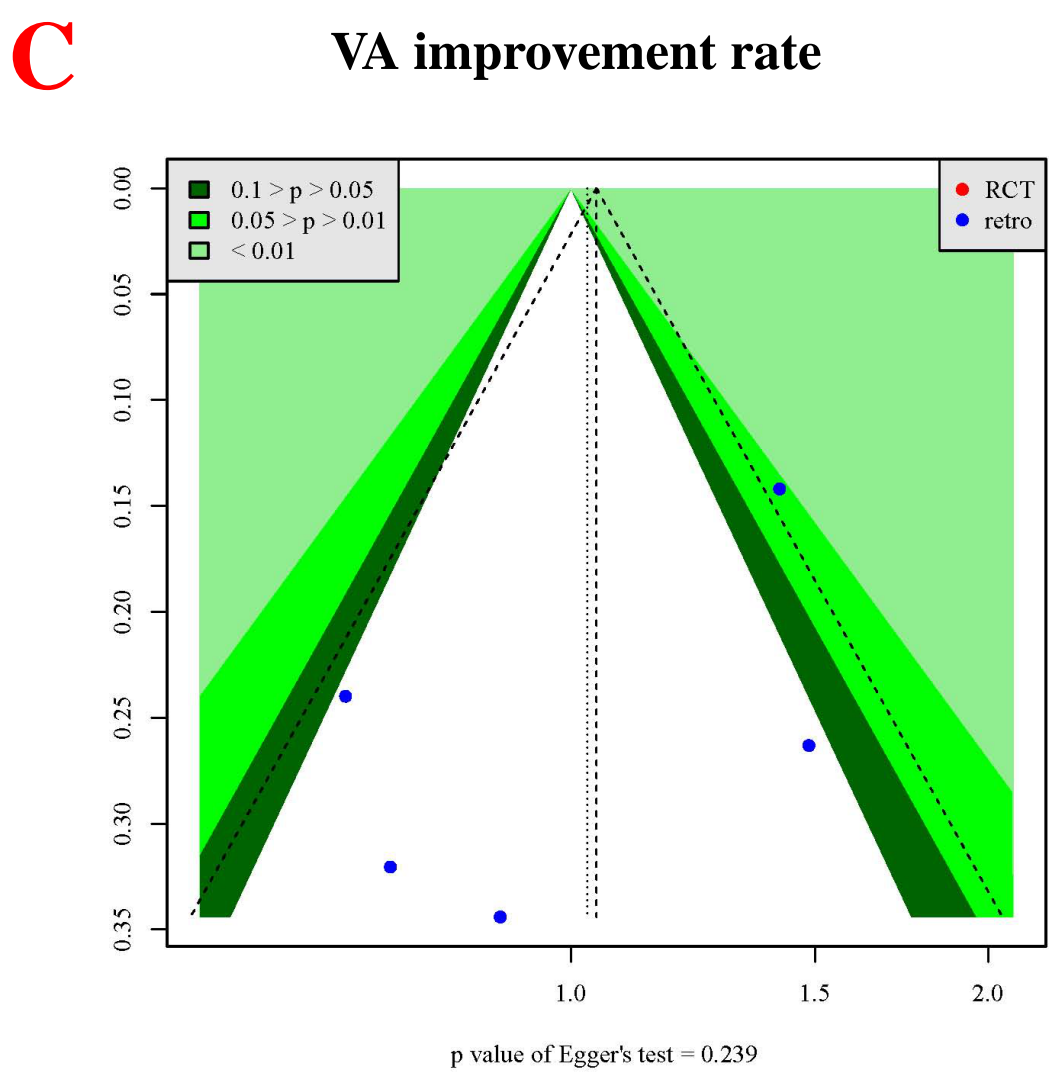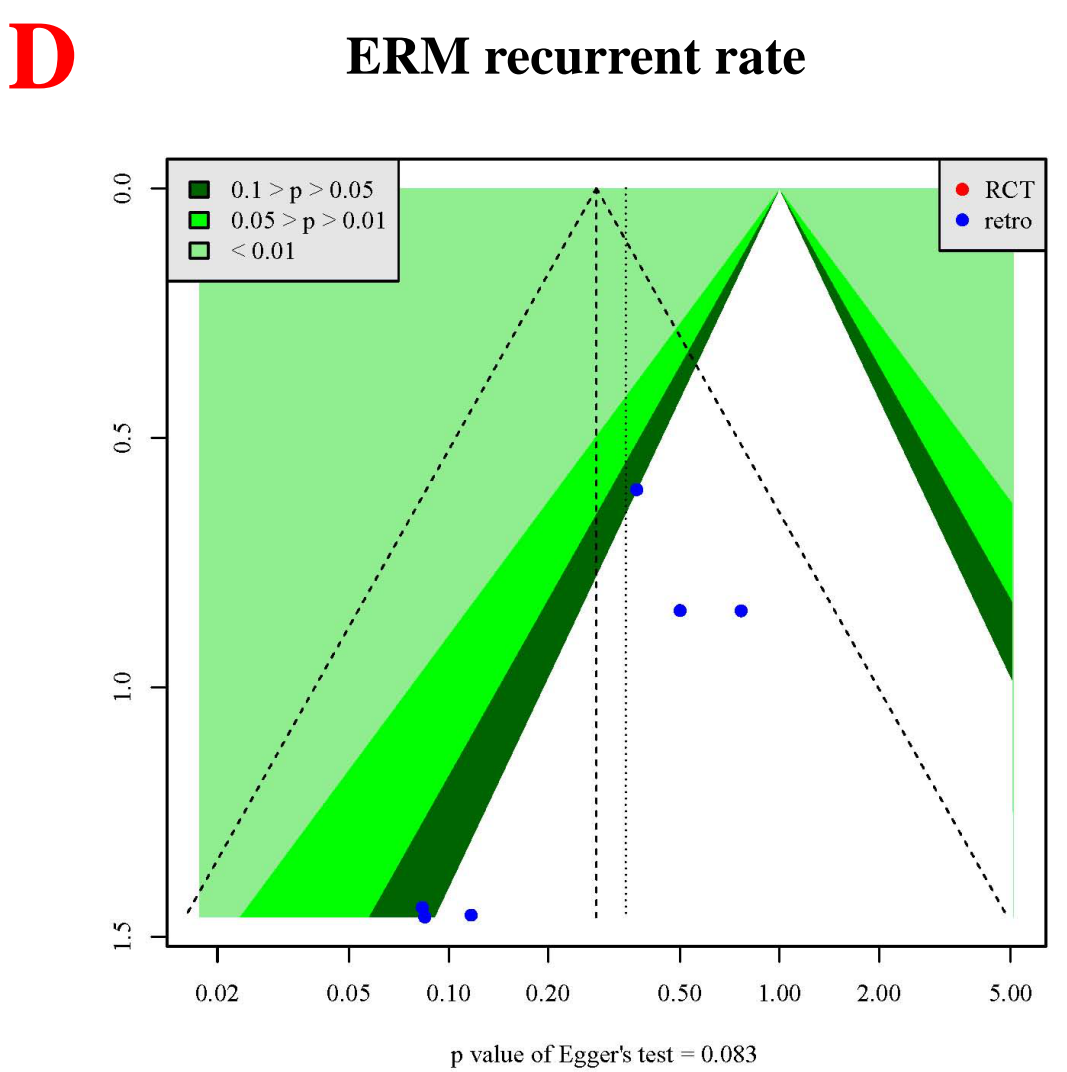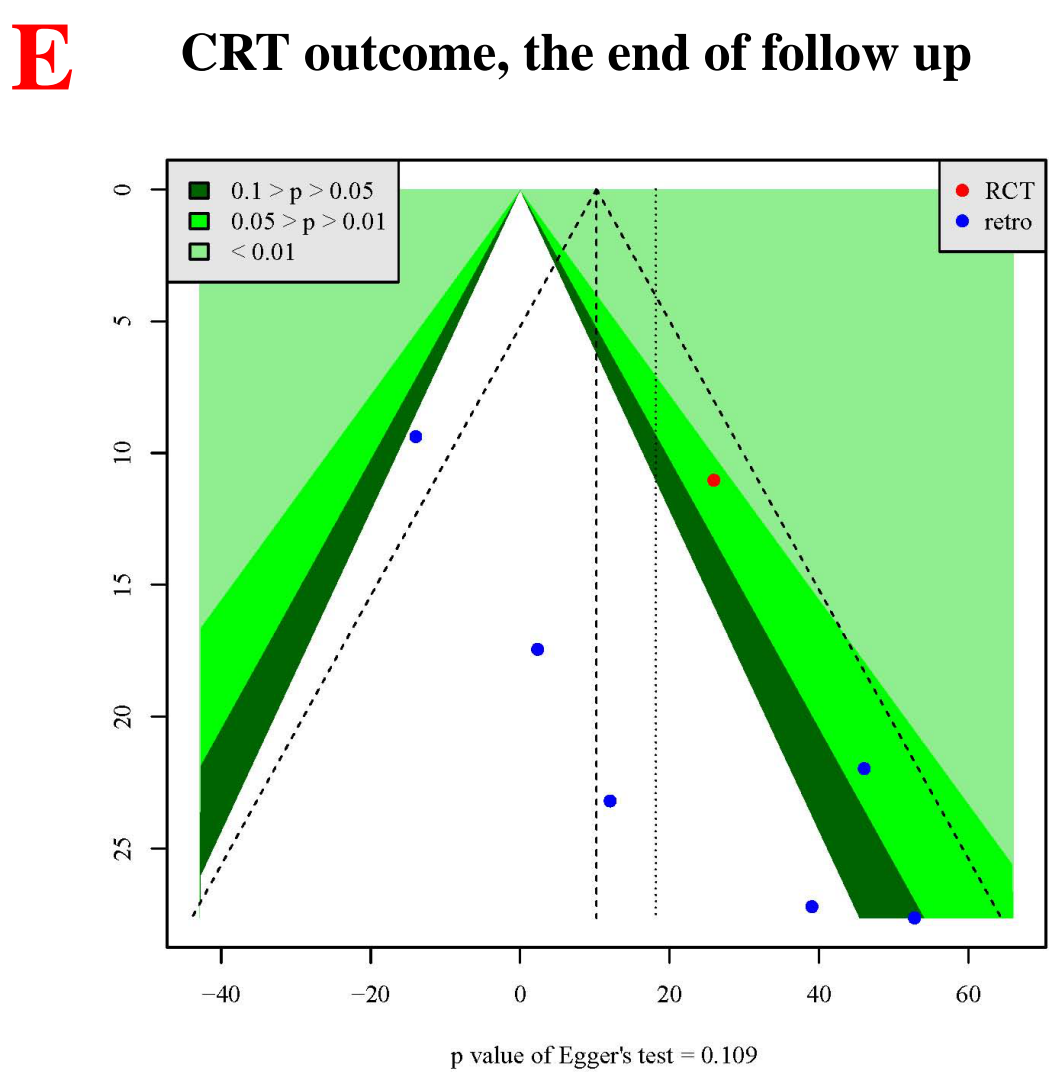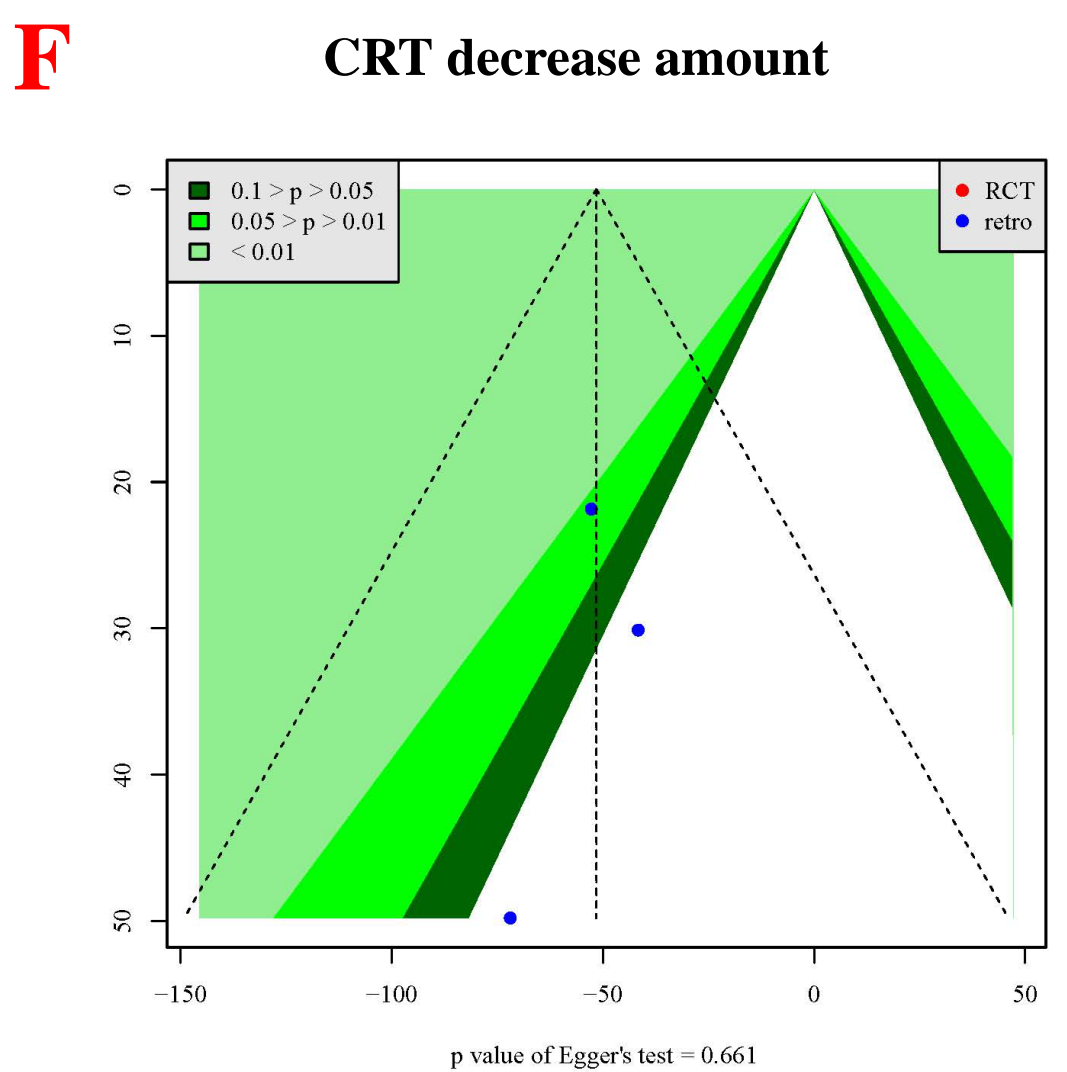

Supplement: S1 Fig — (PDF) [file pone.0179105.s003.pdf]
